# SaludableOmaha: Development of a Youth Advocacy Initiative to Increase Community Readiness for Obesity Prevention, 2011–2012

**DOI:** 10.5888/pcd9.120095

**Published:** 2012-12-06

**Authors:** Leah Frerichs, Jeri Brittin, Catherine Stewart, Regina Robbins, Cara Riggs, Susan Mayberger, Alberto Cervantes, Terry T-K Huang

**Affiliations:** Author Affiliations: Leah Frerichs, Jeri Brittin, Regina Robbins, University of Nebraska Medical Center, Omaha, Nebraska; Catherine Stewart, Independent Consultant, Omaha, Nebraska; Cara Riggs, Omaha South High Magnet School, Omaha, Nebraska; Susan Mayberger, Alberto Cervantes, South Omaha Community Care Council, Omaha, Nebraska.

## Abstract

**Background:**

Childhood obesity rates in minority populations continue to rise despite leveling national trends. Although interventions that address social and environmental factors exist, processes that create demand for policy and environmental change within communities have not been identified.

**Community Context:**

We developed a pilot program in South Omaha, a Nebraska Latino community, based on the community readiness model (CRM), called SaludableOmaha. We used CRM to explore the potential of youth advocacy to shift individual and community norms regarding obesity prevention in South Omaha and to advocate for health-promoting community environments.

**Methods:**

We used CRM to assess supply and demand for health programs, engage the community, determine the community’s baseline readiness to address childhood obesity, and guide youth advocacy program development. We conducted our project in 2 phases. In the first, we trained a cohort of youth. In the second, the youth cohort created and launched a Latino health movement, branded as SaludableOmaha. A third phase, which is currently under way, is directed at institutionalizing youth advocacy in communities.

**Outcome:**

At baseline, the community studied was at a low stage of readiness for change. Our program generated infrastructure and materials to support the growth and institutionalization of youth advocacy as a means of increasing community readiness for addressing obesity prevention.

**Interpretation:**

CRM is an important tool for addressing issues such as childhood obesity in underserved communities because it provides a framework for matching interventions to the community. Community partnerships such as SaludableOmaha can aid the adoption of obesity prevention programs.

## Background

Rates of overweight and obesity have reached epidemic proportions in the United States, and these conditions are associated with a range of physical, social, and economic consequences at both individual and societal levels (1). Of particular concern are childhood obesity rates in minority populations, which continue to rise at a time when the rate of increase in overall national rates appears to be slowing. From 2003 to 2007, the prevalence of obesity among Latino children increased by 24.3% compared with a 7.2% increase among non-Hispanic white children (2).

Although obesity has become a much-studied public health issue, obesity prevention strategies, particularly for youth, have had limited effect on population-level obesity prevalence and trends (3). Research indicates that addressing childhood obesity requires a comprehensive societal approach involving individuals, families, and the community (4). However, interventions to date have focused either on the individual or on environmental and policy factors. Little research has focused on how to create demand for policies, environments, and programs supporting obesity prevention in communities. Youth advocacy may help create a demand for a community response to the childhood obesity epidemic (5,6).

This case study describes the development of a pilot project in an underserved Latino community in Omaha, Nebraska. The project was designed to explore the potential of youth advocacy to increase community awareness and the community’s capacity to generate both the demand for and a supply of community-relevant, health-promoting interventions. The project used the community readiness model (CRM) as a tool to engage the Latino community, assess the community’s readiness for change, and guide training of community youth in advocacy and in advocacy program development. On the basis of our experiences in using the CRM model with this community, we propose a new model to guide future development of a sustainable social movement for childhood obesity prevention.

## Community Context

Omaha, Nebraska, is a growing metropolitan area of more than 865,000 residents (7). Minorities represent nearly 20% of the population, and the Latino population has nearly doubled in the past decade, from 29,250 in 2000 to 56,849 in 2010. During the past 25 years, a large number of Latinos have settled in southeast Omaha (South Omaha), where 55.8% of the population is Hispanic (7).

Poverty rates in Omaha’s Latino population are high; 30% of Latino families with children under age 18 and 55% of families with a single female parent have incomes at or below 100% of the federal poverty guidelines (7). The rates of childhood obesity in South Omaha are similar to national rates in Latino populations. A telephone survey of a random sample of Omaha households found that 31% of adolescents in South Omaha were overweight or obese, compared with 20% in northwest and southwest Omaha, where residents are primarily non-Hispanic white (8). Compared with non-Hispanic white adults, Latino adults in Nebraska have a higher prevalence of obesity (33% vs 25%) and type 2 diabetes (13% vs 7%) (9). These adult health disparities may widen if the high prevalence of Latino childhood obesity persists.

The South Omaha Community Care Council (SOCCC) and the Omaha South High Magnet School (South High) collaborated with the University of Nebraska Medical Center (UNMC) College of Public Health to develop an intervention based on the CRM. SOCCC, a community-based organization whose mission is to provide infrastructure for enhancing community services and community well-being, provided connections to key individuals and organizations in the community. South High provided connections to students and parents. UNMC provided expertise in public health research and obesity prevention.

The goal of our project was to assess the potential of empowering South Omaha’s Latino youth to create a social movement to transform their family and community environments through a community-based effort. We conducted the project in 2 phases. In the first phase, we trained a cohort of youth advocates. In the second phase, Latino youth created and launched a Latino health movement based on the CRM, which they branded SaludableOmaha.

### Assessing community readiness

CRM provided a multifaceted tool to identify gaps in community readiness and capacity, guide strategies for social marketing and youth initiatives, and provide a measure of supply and demand for health to assess future social change. In our study, CRM served as a theoretical framework and method for measuring a community’s readiness to address obesity prevention (10). CRM has been used to address many public health issues in diverse populations by determining the most appropriate program or intervention for a community given its current level of readiness (11–18). Because community needs vary, rarely does 1 size fit all in community-level health promotion and disease prevention strategies (19). CRM addresses community readiness defined by 6 dimensions: presence of community efforts, community knowledge of these efforts, leadership support, community climate, community knowledge about the issue, and availability of resources (20). These dimensions provide a proxy measure of whether a community is demanding change around an issue (ie, measured via leadership support, community climate, and knowledge) and the availability of resources and contextual support (ie, measured via presence of efforts and resources). CRM assesses readiness via semistructured qualitative interviews with key informants, which are scored by using anchored rating scales for each dimension and then averaged for an overall quantitative readiness score that corresponds to 1 of 9 stages (Box). The tool is reliable (reported scorer ratings agree 92% of the time) (20). Our project used CRM to assess the South Omaha Latino community’s baseline level of readiness to address childhood obesity so that the efforts of youth advocates could address the community’s current needs.

Box. Community Readiness Model Stages

**Table:** 

Stage 1. No awareness	Issue is not generally recognized by the community or leaders as a problem (or it may not be an issue).
Stage 2. Denial/resistance	At least some community members recognize that the issue is a concern, but there is little recognition that it might be occurring locally.
Stage 3. Vague awareness	Most feel that there is a local concern, but there is no immediate motivation to do anything about it.
Stage 4. Preplanning	There is clear recognition that something must be done, and there may even be a group addressing the issue. However, efforts are not focused or detailed.
Stage 5. Preparation	Active leaders begin planning in earnest. Community offers modest support of efforts.
Stage 6. Initiation	Enough information is available to justify efforts. Activities are under way.
Stage 7. Stabilization	Activities are supported by administrators or community decision makers. Staff are trained and experienced.
Stage 8. Confirmation/expansion	Efforts are in place. Community members feel comfortable using services, and they support expansions. Local data are regularly obtained.
Stage 9. High level of community ownership	Detailed and sophisticated knowledge exists about prevalence, causes, and consequences. Effective evaluation guides new directions. Model is applied to other issues.

### CRM methods

We identified 2 subgroups of key informants for interviews: community leaders and parents. The South Omaha community partner organizations prepared a list of 36 potential community leaders who were connected to the issue of childhood obesity either by occupation or known general health interest. Project partners worked together to narrow this list to 10 priority organizations that represented sectors considered most relevant and influential regarding childhood obesity, including schools, medical professions, social service organizations, and recreational facilities. Partners also generated a list of potential parent interviewees on the basis of their interest in childhood obesity and health. To qualify for participation, the parent interviewees needed to self-identify as Hispanic or Latino, live in South Omaha, and have children younger than 19.

SOCCC made first contact with potential interviewees and connected them to UNMC study personnel who had been trained to conduct CRM-based interviews for the project. All interviews were completed in person and audio-recorded. The interviewer provided a brief introduction to the project and asked the standard CRM interview questions (20). Although CRM methods recommend 6 interviewees, we interviewed 10 community leaders and 8 parents. Interviews ranged from 20 to 45 minutes. Nine community leaders and 6 parents were interviewed in English; the remainder were interviewed in Spanish.

We transcribed audio recordings verbatim. Two evaluators (UNMC study personnel trained in CRM analysis for the project) scored the interview transcriptions independently. For each parent and community leader interviewed, the 6 CRM dimensions were rated by using the CRM anchored scales. The anchored scales ranged in whole numbers from 1 to 9 for each dimension; 9 represents the most favorable score. The evaluators compared scores and, in the case of disagreement, established reliability by reaching a consensus. To compute the total CRM score for each respondent, the ratings of the 6 dimensions were averaged. A separate CRM score was calculated for community leaders and parents by averaging all respective respondent scores. The stage of readiness was determined for each subgroup of respondents by rounding down the average score to the lower CRM stage (Box). The study was approved by the Institutional Review Board of the University of Nebraska Medical Center (UNMC).

### Youth advocacy program methods

South High provided access to a pool of high school students and their parents who became the initial cohort of youth advocates. Several South High faculty members identified a list of potential applicants with a range of perspectives and skills that the project team defined as important to building infrastructure for the program (eg, graphic arts, media, communication). Project partners held 2 informational events for parents and youth to present the need for obesity prevention, the concept of youth advocacy, a tentative schedule of workshops, and an overview of student expectations. UNMC research team members distributed application forms to youth, and the school collected forms and scheduled interviews. One South High faculty member and a UNMC project consultant completed 15- to 30-minute interviews with all students who applied. A total of 22 students were selected from approximately 40 to 50 applicants; 17 accepted and 14 participated in the program through the first 2 phases. Three dropped out for personal reasons.

During the summer of 2011, youth advocates participated in a series of trainings, workshops, and activities encompassing 2 core phases that incorporated elements of the 6 CRM dimensions. Through this process, we provided youth with advocacy skills and guided them in developing and launching a Latino Health brand (ie, a conceptual framework and corresponding imagery regarding healthy lifestyles that would resonate with the community). In the spring of 2012, we initiated a third phase, which consisted of institutionalizing youth advocacy in the South Omaha community.

Phase 1 involved basic training and consisted of a series of eighteen 4- to 5-hour workshops during 1 month. Workshops included education on obesity, nutrition, and physical activity as well as development of leadership styles, teamwork, and communication. Different formats encouraged active learning (eg, hands-on cooking demonstrations, group discussions). This first phase also engaged youth in brainstorming and creative processes to prepare them for phase 2, in which they developed a unique brand for the movement.

Phase 2, brand development and launch, took place during a 4-week period and guided youth in actively connecting with their community on childhood obesity. On the basis of discussions with youth during Phase 1 regarding their interests and strengths, project facilitators identified potential roles (eg, public relations, creative agency, project coordination) for students. At the end of phase 2, youth developed the infrastructure, framework, and strategies as well as a logo for their Latino health movement, SaludableOmaha. They agreed to create awareness and discuss their proposed strategies with the community during 2 events, a community-wide health fair and a formal dinner for community leaders and parents.

During spring 2012, SaludableOmaha moved into phase 3, institutionalization. During this phase a cohort of new youth advocates became involved and successfully led a school-based healthy eating initiative that demonstrated potential for the movement to grow. The project was selected by youths, given the low readiness of the community as a whole, to increase awareness in their school environment. South High began to institutionalize the youth advocacy program in its regular curricula and developed in parallel a local partnership with Live Well Omaha Kids (LWOK) (21), the youth component of a community-based coalition for greater Omaha (www.livewellomaha.org). This partnership resulted in a natural collaboration of 2 youth groups: 1) LWOK Youth Advisory Committee , comprising student leaders representing high schools throughout Omaha, and 2) South High Character in Action, a class to engage students in community service activities leading the SaludableOmaha effort. Together, the groups began Green is Go, an initiative to advocate for no- to low-cost changes to the South High cafeteria to improve eating habits. Because evidence suggests that social networks influence health and health behaviors (22), the Green is Go initiative uses social marketing and media. For example, students plan to post pictures and stories (eg, images of lunch trays with healthful foods) on the SaludableOmaha Facebook page for peer-to-peer networking and discussion.

## Outcomes

### CRM outcomes

We determined that the South Omaha community is at a low stage of readiness to address childhood obesity. Community leaders scored at stage 3, “vague awareness,” indicating they felt childhood obesity was a local issue, but there was no significant effort to take action within the community. Parents scored at stage 2, “denial/resistance,” indicating that they felt that although some people identify childhood obesity as a problem, the community as a whole does not recognize it as a concern.

The average dimension scores were consistent (Table). The average score ranged between 2 and 3 for both community leaders and parents with the exception of the “efforts” dimension, for which the score was higher. Participants indicated that many different types of nutrition- and physical activity-related programs and strategies were available. However, none specifically targeted childhood obesity or appeared effectively coordinated across the community.

**Table:** Average Community Readiness Model (CRM) Scores^a^, SaludableOmaha, 2011–2012

CRM Dimension^b^	Community Leaders (n = 10)	Parents (n = 8)
Efforts	6.9	4.8
Knowledge of efforts	3.3	2.8
Leadership	3.2	2.1
Community climate	2.6	2.4
Knowledge of issue	2.2	2.4
Resources	3.4	3.0
CRM stage **c**	3	2

Abbreviations: MS, veterans with multiple sclerosis; GV, general veteran population; GP, general population.

a Scores are calculated for each dimension by averaging anchored rating across all interviews. The anchored scales range in whole numbers from 1 to 9 for each dimension; 9 represents the most favorable score.

b Anchored rating scales are defined for 6 dimensions of readiness identified by the CRM (10) and used to score each interview.

c The average score across dimensions, rounded down, corresponds to the overall community readiness stage.

### Youth advocacy program outcomes

The youth advocacy component of this project is intended to create upward momentum in the South Omaha community through the levels of readiness to address childhood obesity. Although community ownership is critical to the long-term success of community programs, it has been more the exception than the rule to involve youth directly in the design and implementation of programs (5). By using a youth-driven approach, the SaludableOmaha project generated community-relevant solutions and improved sustainability by creating a new generation of community activists to champion the cause long-term.

The initial phases of the youth advocacy program created the framework necessary to support a sustainable childhood obesity program to address the low levels of community readiness. The youth generated a culturally and locally relevant brand that can be used to market and promote efforts across the community. Furthermore, the initial workshops allowed the development of appropriate community relationships that led to an institutionalization process that can be used as a vehicle for continued youth-led initiatives.

### An integrated model for generating social movement with youth advocacy

The experiences of implementing this youth advocacy intervention allowed us to develop a model to guide future research and programming (Figure). This model can be used to generate knowledge of the potential underlying mechanisms to create social movement using youth advocacy as a central approach. A youth advocacy approach is placed at the center of the model, and includes horizontal integration (ie, support from community partners for local growth) and vertical integration (ie, linkage to national networks for diffusion to additional communities) to create a health movement to address childhood overweight and obesity at a local level.

**Figure:**
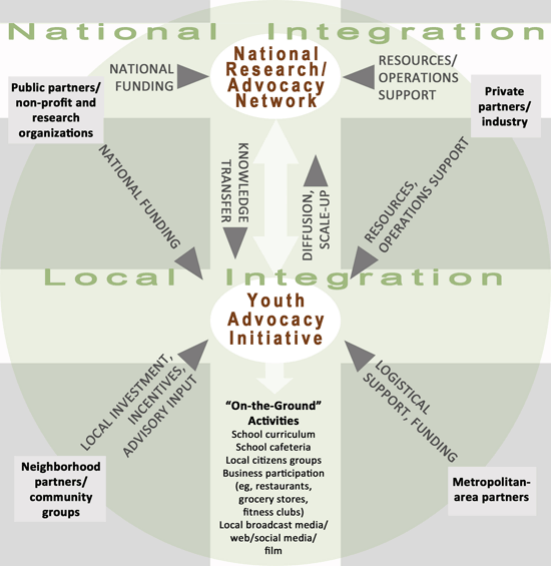
Proposed model for a horizontally and vertically integrated social movement, based on the experiences of SaludableOmaha, to guide further research on the potential underlying mechanisms of such a social movement.

Horizontal integration involves support from community groups and metropolitan-area partners to assist youth in engaging in “on-the-ground” activities (eg, Green is Go). The local partners provide various types of support; however, local investment (eg, school in-kind support of space) and logistical support from metropolitan-area partners (eg, connection to nutrition expertise) are vital. Vertical integration via linkage to state, regional, and national networks supported by public and private partners could expand the effort locally and create pathways to diffuse the movement to additional communities. Vertical integration also includes various types of support at both national and local levels (eg, research funding for a centrally coordinated national network and local chapters). Private partners’ operational support is also instrumental for growth (eg, communications firms could be recruited as volunteers to maintain social media and marketing).

## Interpretation

CRM is an important tool for addressing issues such as childhood obesity in underserved communities because it provides a framework for matching interventions to the community. South Omaha was at a low level of readiness for change, which is not uncommon in minority communities where mainstream resources and communication fail to reach and engage stakeholder groups. Although CRM interviewees described numerous nutrition- and physical activity-related programs (eg, soccer clubs, food banks), none specifically addressed obesity prevention, which indicates a gap in programming that actively targets individuals at risk or systematically addresses the complex factors of obesity prevention. Thus, efforts that do not consider readiness for change and tailor strategies to stage or readiness will likely neglect communities with lower readiness but higher need.

The CRM is also an effective way to encourage community ownership. The knowledge gained through interviews provides a platform for generating discussion about needs and priorities integral to creating change. In our project, the CRM interviews also helped project partners learn about the community and established relationships that guided progress (eg, community leader interviewees have been assisting with youth-led efforts in Phase 3).

The CRM also helped inform youth advocacy program and strategies. At the end of Phase 2, participating youth planned 2 activities, both designed for low levels of community readiness and focused on increasing awareness of overweight and obesity. Youth also identified social marketing as important for addressing low readiness. In Phase 3, enhanced marketing through social media tools such as Facebook are in development. These strategies have potential for increasing engagement of younger populations.

Several challenges are inherent in youth advocacy efforts, and many questions remain unanswered regarding best practices (6). Youth in South Omaha were largely disconnected from community health leaders and were unfamiliar with the concept of leadership. Thus, projects that focus on enhancing youth’s leadership ability in addressing social issues are needed, but such projects also require more time and development to effectively address the disconnection. Youth participation was key to SaludableOmaha’s cultural and social relevance. Youth directly developed their own brand and strategies based on their perceptions, attitudes, and experiences. For example, youth painted a mural in the community neighborhood with relevant cultural themes to depict the need to improve health. However, the continued growth and sustainability of the youth advocacy effort requires a process for continuing to engage youth (ie, new freshman cohorts) who may have different interests and skills to address evolving needs.

The low level of readiness that the CRM assessment showed reinforces the need for an intervention that could be tailored to community readiness. The objective of this pilot youth advocacy effort was to explore possible mechanisms that encourage ongoing growth and sustainability of health-promoting environments and behavior change. Thus far, this effort created an infrastructure for communities to address issues as they evolve at various levels. South High’s support of SaludableOmaha and key connections and resources were vital to the program’s success and show potential for the future sustainability of the effort as it is incorporated in regular school programs. However, support and resources are needed for sustainability and continued growth.

Youth advocacy for obesity prevention is a new area, and this pilot project is one of the first to illustrate a potential mechanism to promote normative change at the community level. The horizontal and vertical integrated model we propose shows how local expansion and outward growth create a framework for environmental change and shifts in cultural and social norms that can be locally tailored but also broad-reaching. Our proposed model needs further testing and development. For example, quantifiable measures are needed at multiple levels to monitor peer-to-peer effects and community trends such as social media network analysis and policy changes in community institutions. The CRM also provides a useful measure of social change. This continued research will enhance our ability to understand and develop interventions that improve alignment of community demand with the supply of health-promoting environments and policies.
